# Study Protocol for Mindfulness-Based Yoga Versus Physical Exercise on the Psychological Well-Being in Students With Early Visual Impairment: A Three-Armed, Multi-Centered, Randomized Controlled Trial

**DOI:** 10.7759/cureus.69240

**Published:** 2024-09-12

**Authors:** Danqing Li, Soubhagyalaxmi Mohanty, Ramesh Mavathur, Vijaya Y Vageesh, Anup Jain, Arun Gopi, Nagarathna Raghuram

**Affiliations:** 1 Yoga and Humanity Division, Swami Vivekananda Yoga Anusandhana Samsthana (S-VYASA) University, Bangalore, IND; 2 Yoga and Life Science Division, Swami Vivekananda Yoga Anusandhana Samsthana (S-VYASA) University, Bangalore, IND; 3 Physiology, Jagadguru Sri Shivarathreeshwara (JSS) Medical College and Hospital, Jagadguru Sri Shivarathreeshwara (JSS) Academy of Higher Education and Research, Mysore, IND; 4 Community Medicine, Jagadguru Sri Shivarathreeshwara (JSS) Medical College and Hospital, Jagadguru Sri Shivarathreeshwara (JSS) Academy of Higher Education and Research, Mysore, IND; 5 Preventive Medicine, Swami Vivekananda Yoga Anusandhana Samsthana (S-VYASA) University, Bangalore, IND

**Keywords:** early visual impairment, physical exercise, psychological wellbeing, quality of sleep, yoga research

## Abstract

Background

People with visual impairment (VI) tend to face more psychological distress than normally sighted individuals due to mobility restrictions, fear of falling, and sleep disturbances. However, research to address these problems is rare. This study aims to investigate the effect of mindfulness-based yoga versus physical exercise on the psychological well-being of individuals with VI.

Methods

This study will be a single-blinded, three-armed, multicentered, randomized controlled trial (RCT). A total of 132 participants with VI (ages 15-25) will be recruited in the study and will be randomly assigned to either group 1 (mindfulness-based yoga), group 2 (physical exercise), or group 3 (wait-list control). Groups 1 and 2 will receive intervention for 40 hours (eight weeks, weekly five days, one hour/day), whereas group 3 will continue their daily activities as usual. The intervention will take place in the afternoon from Monday to Friday. The timing varies between 4-5 pm according to the different time schedules of the institutions of the blind. Three times, assessments will be conducted at T0 (baseline), T1 (eighth week at the completion of the intervention), and T2 (sixth month following the completion of the intervention). ANOVA will be used to find out the differences between groups; repeated measures ANOVA will be used to check within-group changes.

Trial status

The study was first screened in December 2021. The recruitment of participants has been completed in two centers covering 62 individuals with VI, and intervention started in August 2022. The data collection is still ongoing due to the nature of the study design, a specific demographic, complex logistics, and administrative bottlenecks. The study incorporates three different groups and a substantial sample size (n=132). The specific demographic, people with visual impairments, are rare and difficult to locate. In addition, a six-month follow-up assessment contributes to complex procedures while coordinating between various institutions and securing necessary authorizations.

Discussion

This study will be the first comprehensive RCT to investigate the psychological well-being of the VI population with various psychophysiological and hormonal parameters in multiple centers. The presence of physical exercise and a wait-list control group will further elucidate the potential mechanism of Mindfulness-based yoga. Mindfulness-based yoga can be integrated into educational and rehabilitation systems to enhance the well-being of individuals with VI.

## Introduction

People with visual impairment (VI) face a higher risk of psychological distress, which affects their multidimensional health aspects [1]. The mortality rate among the visually impaired is much higher than that of normally sighted people [2]. Visual impairment, characterized by low vision or blindness, is a functional limitation of the eyes. At least 2.2 billion people worldwide are visually impaired, and this is gaining growing concern in the development of public health policies [3]. Based on global population growth and increased life expectancy, it is estimated that global blindness will triple and moderate-to-severe VI will double by the year 2050 [4]. Though VI affects hundreds of millions globally, 90% of those with VI live in low- and middle-income countries [5]. India contributes 8 million people with blindness and 62 million with VI to the global population [6]. 

Loss of vision affects various aspects of life, such as reduced physical activities, unintentional injuries, loneliness, disturbed sleep, and low self-esteem [7-12]. Compared with the general population, people with VI are at a higher risk of experiencing psychological distress, such as anxiety and depression [13,14]. In return, the accumulated stress can lead to more vision loss [15]. Various factors contribute to psychological distress in the visually impaired, such as poor social communication, compromised daily role functions, and self-care capacities [16-19]. In addition, they face a higher chance of unemployment and more stress from family responsibilities and marriage expectations than their sighted peers [20-22]. Psychological distress in VI induces fatigue, low self-esteem, caregiver distress, and socioeconomic burden at large [23-26]. In addition, people with VI, especially those with no perception of light, tend to experience poorer sleep quality than the sighted due to the desynchronized rhythm of melatonin secretion [11]. Sleep disturbance is a mediating factor in mental health, and sleep management has been used as an effective strategy to treat mental disorders [27].

Even though individuals with VI encounter a high incidence of mental distress, the challenge they face is hardly noticed and barely resolved [1,28]. The pharmacological management of anxiety, depression, and insomnia is complicated because of its adverse effects, as antidepressants might provoke agitation, nervousness, suicidal thoughts, and even reduce eyesight in partially blind [29]. Hence, adopting a complementary and alternative management (CAM) approach to address the adverse and limited effects of pharmacological management is essential. It has revealed its positive impact on reducing negative emotions and stress hormones and increasing self-control, positive emotions, sleep quality, and immune reactivity [30-32]. Mind-body exercise is one of the most extensively practiced non-pharmacological treatments for its gentle and low-risk nature [33-35]. Yoga, promoted by the United Nations and welcomed by the whole world [36], has a beneficial impact on physical outcomes in the population with VI [37,38]. Mindfulness-based yoga (MY) integrates mindful awareness into traditional yoga practices, combining mindful awareness to enhance self-awareness, focus on the present moment, and cultivate a non-judgmental attitude by paying attention to bodily sensations, thoughts, and emotions [35]. Apart from that, MY encourages practitioners to extend the practice of being present beyond yoga mats into their daily lives, creating a profound impact on their overall well-being [39]. Mindfulness-based yoga is a valuable component of complementary and alternative management, providing a holistic approach to a sense of well-being by blending the physical benefits of yoga with the mental, emotional, and spiritual benefits of mindful awareness.

Most of the previous research has explored populations with VI who are either middle-aged or old adults (above 45 years) or children (below 16 years) [37,38]. However, adolescents and young adults (15-25 years) with VI have largely been neglected despite facing unique challenges in academics and complex life situations [40,41]. This age group contends with pressures related to future job opportunities, relationships, and academic performance [42-44]. Mindfulness-based yoga, a practice aimed at mastering mind fluctuations, offers a profound solution for these challenges, starting from physical postures (asana), merging through breath regulation (pranayama), and reaching a sense of wellness and a peaceful state of meditative mind (dhyana). Mindfulness-based yoga nourishes inner well-being and resilience, helping this age group to manage stress effectively [45,46]. Therefore, to fill the research gap, this study will investigate the effects of MY in addressing psychological distress in adolescents and young adults with early VI. To enhance the internal validity, some participants will be randomly assigned to the physical exercise (PE) group since it only focuses on the physical dimension of well-being. The dosage of PE will be the same as that administered in the MY group.

## Materials and methods

**Figure 1:**
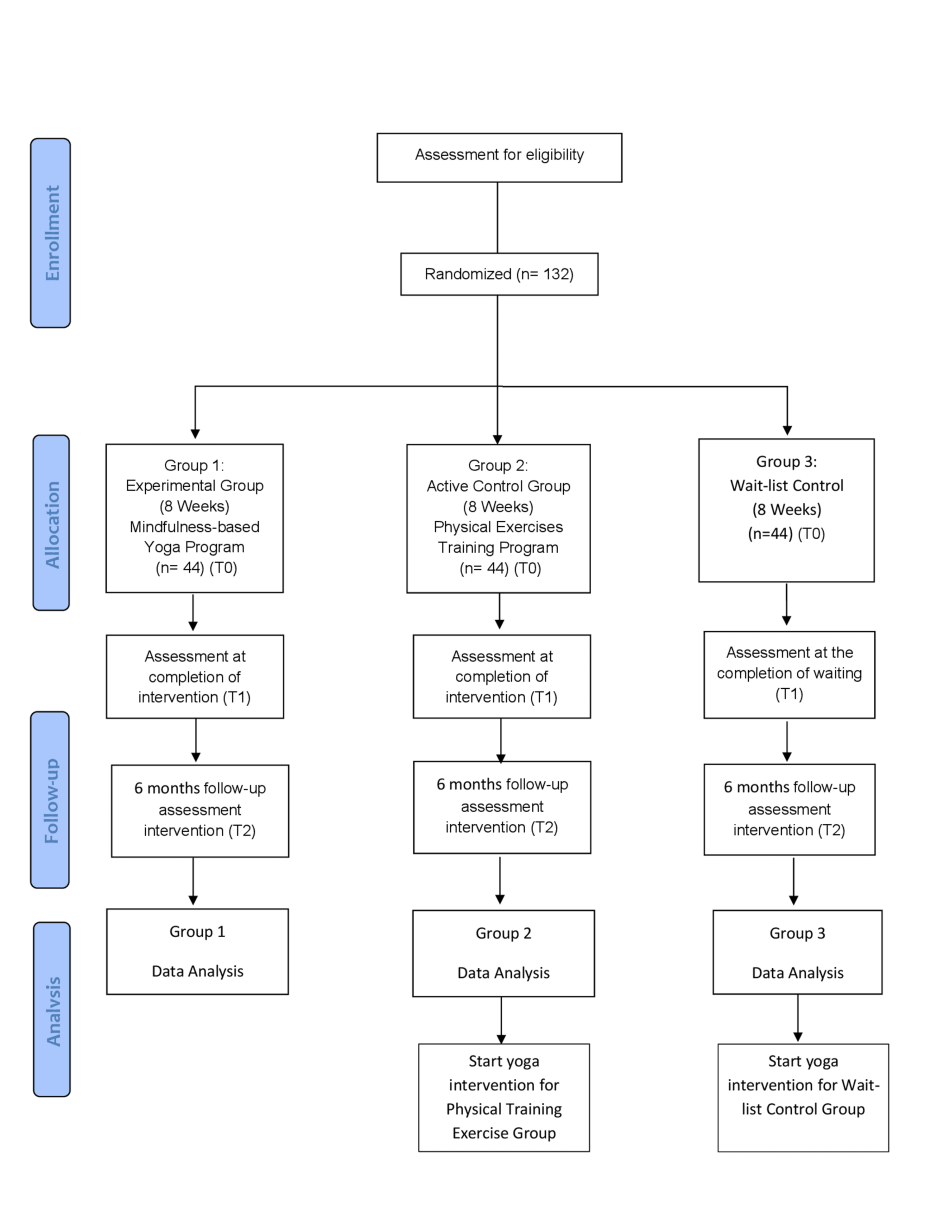
Consolidated Standards of Reporting Trials (CONSORT) flow diagram of the study T0 - baseline; T1 - at the perception of intervention (eight weeks); T2 - six months following the intervention

A single-blinded randomized controlled trial (RCT) will examine the effects of MY versus PE to investigate the psychological well-being, physiological well-being, physical well-being, social well-being, spiritual well-being, vision-related quality of life, and sleep quality of students with early VI. This trial is designed according to the Consolidated Standards of Reporting Trials (CONSORT) statement (Figure 1) and reported according to the Standard Protocol Items: Recommendations for Interventional Trials (SPIRIT) statement (Table 1).

**Table 1:** Standard Protocol Items: Recommendations for Interventional Trials (SPIRIT) HADS,- Hospital Anxiety and Depression Scale; OHQ - Oxford Happiness Questionnaire; ECG - electrocardiograph; HRV - heart rate variability; RR - respiratory rate; GSR - galvanic skin resistance; EMG - electromyography; TUG - Time Up and Go Test; 30CST - 30-second Chair Stand Test; MSPSS - Multidimensional Scale of Perceived Social Support; HWS - Holistic Well-being Scale; VisQoL - Vision Quality of Life Index; PSQI - Pittsburgh Sleep Quality Index; T0 - baseline; T1 - at the completion of intervention (eight weeks); T2 - six months following the intervention

	Enrolment	Allocation	Post-allocation			
Timepoint	0	0	T0	Intervention	T1	T2
Enrolment						
Eligibility screen	X					
Ophthalmology test		X				
Oral Informed consent with fingerprint	X					
Allocation		X				
Interventions						
Mindfulness-based yoga				X		
Physical exercise				X		
Wait-list control				X		
Assessment						
Sociodemographic characteristics			X			
HADS, OHQ, biomarkers, ECG, HRV, RR, GSR, EMG, TUG, 30 CST, MSPSS, HWS, VisQoL, PSQI			X		X	X
Treatment-related Information: adverse events, adherence to assigned treatment			X		X	X
Potential confounders: food			X		X	X

Objectives and hypothesis

The objectives will be as follows: to investigate the effect of mindfulness-based yoga (MY) on 1) psychological well-being, 2) physical well-being, 3) physiological well-being, 4) spiritual well-being, 5) health-related quality of life, and 6) sleep quality in individuals with early moderate-to-severe visual impairment and blindness.

We hypothesize that 1) the MY program will effectively improve psychological well-being, physiological well-being, physical well-being, social well-being, spiritual well-being, vision-related quality of life, and sleep quality in individuals with early VI, and 2) the MY will be superior to PE in improving psychological well-being, physiological well-being, physical well-being, social well-being, spiritual well-being, vision-related quality of life, and sleep quality in individuals with early VI.

Design

This study is a multi-centered, assessor-masked, three-armed RCT. A convenient sampling method will be adopted for the selection of participants. They will undergo the ophthalmologic visual acuity test for screening. Eligible participants will be randomly assigned to either MY, PE, or wait-list control group through computer software (Randomizer) based on gender, age, and severity of vision loss, in which each group contains the same proportion of the factors mentioned above.

The intervention will be for eight weeks (one hour/day, five days a week), and a follow-up assessment will be carried out six months after the completion of the intervention. The study contains three times evaluations in which the baseline assessment (T0) will be before group division. Post-intervention assessment (T1) will be at the end of the intervention for eight weeks. Follow-up assessment (T2) will be six months after the completion of the intervention. The principal researcher will make unannounced weekly visits to the intervener's class to assess the adherence, consistency, and quality of the intervention. In each classroom, MY or PE, two teaching assistants will support the main instructor in conducting the classes to minimize the risk of injury, such as ankle and knee injuries for the participants.

Randomization method

Stratified randomization will be employed to randomly assign participants based on three factors: age, gender, and the severity of visual impairment. This approach aims to reduce potential confounding effects and enhance the validity of the results.

Participants will first be categorized by gender into two groups: male and female. Within each gender group, participants will be further subdivided into two age categories: adolescents (15-17 years) and young adults (18-25 years). Each of these subcategories will then be divided according to the severity of visual impairment: 1) Mild visual impairment: visual acuity worse than 6/12 but better than or equal to 6/18); 2) moderate visual impairment: visual acuity worse than 6/18 but better than or equal to 6/60); 3) severe visual impairment: visual acuity worse than 6/60 but better than or equal to 3/60); 4) Blindness visual impairment: visual acuity worse than 3/60 or a corresponding visual field loss to less than 10 degrees in the better eye with the best possible correction) [47]. Participants in each subgroup at this third level will have their names entered into a computer software program called Randomizer for shuffling. After shuffling, participants will be randomly allocated to one of three groups: the mindfulness-based yoga group, the physical exercise group, or the wait-list control group. The allocation ratio will be 1:1:1. 

Participants recruitment

People with moderate-to-severe VI will be recruited through two primary sources, namely two non-profit rehabilitation organizations (NGOs): the Samarthanam Trust for the Disabled and the Devnar Foundation for the Blind. The sample population will be drawn from the two above-mentioned NGOs by adopting the random sampling method. The intervention will be conducted across multiple sites nationwide, as participants with VI are taken care of by various branches of these two NGOs.

Potential participants will be approached through coordinated efforts with the institutions for the blind. Information sessions will be organized and conducted with the authorization of the governing body and instructors to explain the study's objectives, procedures, and potential benefits. Detailed written materials will be provided, and verbal explanations will be given to ensure comprehension. Participants will have the opportunity to ask questions and discuss any concerns. Informed consent will be obtained from each participant before inclusion in the study, ensuring they fully understand their involvement and the study's requirements. The research will not involve professors or instructors of the visually impaired participants to avoid any perception of obligation among students to participate.

Inclusion and exclusion criteria

Inclusion criteria include the following: 1) a clinical diagnosis of early VI, with a visual disability severity rating of moderate-to-severe VI according to WHO International Disease Classification 11 Distance Visual Impairment [48]; 2) age between 15-25 years old; 3) early visual impairment, early VI refers to people who have been blind since birth or early childhood (<5 years), whereas late VI indicates persons who lost sight after learning to read print [49]; (4) participants who are willing to give oral consent or assent along with a thumbprint. For participants under 18 years old, a consent form signed by their legal guardian will be needed as well.

Exclusion criteria are the following: 1) participants who are receiving treatment for mental disorders currently or with uncontrolled mood disorders; 2) current participation in any other pharmacological trial or cognitive behavioral program; 3) cognitive impairment such as mental retardation; 4) other debilitating medical conditions except VI, such as having a hearing impairment, epilepsy, or physical disability (e.g., deformity of limbs), that can impede full participation in the study; 5) having a secondary VI, such as Grave's ophthalmopathy (thyroid eye disease), Bechet syndrome (blood vessel inflammation throughout the body), uveitis (inflammation of the middle layer of the eye), multiple sclerosis (autoimmunity), in which symptoms are caused by a different system disorder or another illness [23].

The inclusion criteria are to maximize the enrolment of appropriate participants, while the exclusion criteria are used to screen out participants who are with medical conditions for which yoga or exercise is contraindicated, which pose a safety risk, preclude fully informed consent, or hinder compliance with interventions (e.g., mental retardation).

Sample size

A previous study on children (9-16 years) with VI reported a moderate effect size of 0.4 for overall well-being [37]. To anticipate a similar effect size of at least 0.4 on adolescents and young adults (15-25 years) and using the software G Power 3.1 with 80% power at a 5% level of significance, 36 subjects will be in each arm of the study. Considering an attrition rate of 20%, we will recruit 132 subjects, with 44 per arm.

Interventions

The MY and PE programs will be led by qualified professionals with at least 10 years of teaching experience. All instructors are certified by recognized institutions and have completed a specialized one-week training in adaptive techniques for visually impaired participants conducted by the principal investigator. This training covers a specialized teaching methodology for visually impaired individuals, comprehensive safety precautions during practices, and a deep understanding of physical recreation.

Both MY and PE interventions will take place at the participants' institutes as an add-on course to their daily extracurricular activities. The intervention will span eight weeks, with sessions held Monday to Friday for one hour between 4-5 pm, although the exact timing may vary based on each institute's schedule. Each class will include approximately 15 participants, ensuring sufficient individual attention. Instructions will be delivered verbally, with hands-on assistance provided as needed to ensure all participants can perform the movements correctly.

The chosen duration of eight weeks for the intervention (five days per week) is based on well-established research indicating that this timeframe is optimal for observing significant psychophysiological changes in participants [39]. Consistent daily practice over this period allows for the accumulation of benefits while maintaining participant engagement and adherence. The frequency ensures regular and sustained exposure to the intervention, which is crucial for measurable outcomes.

Group 1: Mindfulness-Based Yoga Group

The MY program, specifically designed for individuals with VI, is developed based on findings from two previous studies [38,50]. This comprehensive yoga module features a structured sequence that begins with a warm-up, yoga postures (asana), breathing exercises (pranayama), and meditation (dhyana). The program integrates mindfulness-based meditation and yogic sleep (Yoga Nidra), emphasizing mindful awareness throughout each session. To ensure clarity and accessibility, asanas and pranayama will be taught using auditory instructions and tactile feedback. Meditation and yoga Nidra sessions will be enhanced with light background music to foster relaxation and focus. This approach is crafted to maximize the therapeutic benefits of yoga for participants with VI. The detailed yoga module is outlined in Table 2, providing a clear framework for the intervention.

**Table 2:** Overview of mindfulness-based yoga program for the visually impaired Each day of the week is dedicated to a different yoga practice module.

Day	Monday	Tuesday	Wednesday	Thursday	Friday
Theme for meditation	Awareness of interconnection body, breath and mind	Steadiness, purity of body and mind	Sleep management	Gratitude and contentment	Love and compassion
Warm-up (10 min)	Sectional breathing (chest, abdomen, and clavicular) jogging and jumping forward bending backward bending body twisting	Full yogic breathing jogging and jumping hip rotation knee rotation	Ujjayi pranayama (psychic breath) rotation of full body joints: feet rotation, toes rotation, spinal rotation, wrist rotation, shoulder rotation, neck rotation.	Bastrika pranayama (Bellows breathing) hand movement with breath in sitting pose	Kapalabhati (frontal brain cleansing breath), Marjaryasana (cat pose), Bitilasana (cow pose), Shashankasana (child pose)
Yoga Asana (30 min)	Standing: Ardha Chakrasana (half wheel pose), Padahastasana (hand to foot pose), Trikonasana (triangle pose); sitting: Vajrasana (thunderbolt pose), Paschimottanasana (back stretching pose); prone: Shalabasana (locust pose), Bhujangasana (cobra pose), Makarasana (crocodile pose); supine: Pavan Muktasana (knee to chest pose), Setubandhasana (bridge pose), Shavasana (corpse pose)	Standing: Tadasana (mountain pose), Trikonasana (triangle pose), Parivritta Trikonasana (twisted triangle pose); sitting: Vakrasana (half spinal twist pose), Ardha Matsyendrasana (half fish lord pose), Baddakonasana (butterfly pose); prone: Shalabasana (locust pose), Bhujangasana (cobra pose), Dhanurasana (bow pose); supine: Pavan Muktasana (knee to chest pose), Sarvangasana (shoulder stand), Matsyasana (fish pose)	Standing: Utkatasana (chair pose), Vriksasana (tree pose), Virabhadrasana 1 (warrior pose 1); sitting: Naukasana (boat pose), Janushirshasana (head to knee pose); Paschimottanasana (back stretching pose); prone: Shalabasana (locust pose), Bhujangasana (cobra pose), Shashankasana (child pose), supine: Naukasana (boat pose), Setubandhasana (pridge pose), Natrajasana (lord shiva pose)	Twelve poses of Surya Namaskar: Pranamasana (prayer pose), Hasta Utthanasana (raised arms pose), Padahastasana (hand to foot pose), Ashwa Sanchalanasana (equestrian pose), Parvatasana (mountain pose), Ashtanga Namaskar (salute with eight parts), Bhujangasana (cobra pose), Parvatasana (mountain pose), Ashwa Sanchalanasana, (equestrian pose), Padahastasana (hand to foot pose), Hasta Utthanasana (raised arms pose), Pranamasana (prayer pose); supine: Shalabasana (corpse pose)	Standing: Padangushthasana (hand to big toe pose), Parshvakonasana (extended side angle pose), Parivritta Parshvakonasana (revolved side angle pose); sitting: Dandasana (staff pose), Paschimottanasana (back stretching pose), Purvottanasana (upward plank pose), Marichyasana (dedicated to Marichi); prone: Bhujangasana (cobra pose), Dhanurasana (bow pose), Makarasama (crocodile pose); supine: Pavan Muktasana (knee to chest pose), Setubandhasana (bridge pose), Shavasana (corpse pose)
Pranayama (10 min)	Nadi Shodhana Pranayama (psychic network purification), Brahmari Pranayama (humming bee breath)	Ujjayi Pranayama (psychic breath), Nadi Shodhana Pranayama (psychic network purification)	Sheetali Pranayama (cooling breath) Bramari Pranayama (humming bee breath)	Nadi Shodhana Pranayama (psychic network purification)	Ujjayi Pranayama (psychic breath) Bramari Pranayama (humming bee breath)
Mindfulness practice (10 min)	Mindfulness of body, breath, and mind meditation	Mindfulness of sound to silence meditation & aura meditation	Yoga Nidra (yogic sleep)	Mindfulness of contentment meditation	Mindfulness of loving kindness meditation

Group 2: Physical Exercise Group

The development of the PE module is based on two prior research concerning physical exercise for individuals with visual impairments [51,52]. This module is of a lower cardio intensity, considering that it should be compatible with individuals with VI in terms of intensity and comparable to the experimental group. The method of teaching PE will adopt a similar teaching method - audial and tactile input - so that participants will be able to understand the instructions clearly. The PE module is illustrated in Table 3.

**Table 3:** Overview of physical exercise intervention for the visually impaired Each day of the week is dedicated to a different physical exercise practice module. Warm-up: 10 seconds 2 sets with 10 seconds of rest; Main workout: 20 seconds 2 sets with 20 seconds of rest; Upper lower body: 10 reps with 3 sets with 30 seconds of rest; Cool down: 30 seconds 2 sets with 10 seconds of rest

Day	Monday	Tuesday	Wednesday	Thursday	Friday
Theme	Strength and endurance	Upper body	Core	Strength and endurance	Lower body
Warm-up (15 min)	Neck rotation, shoulder rotation, waist rotation, ankle rotation, wrist rotation, spot skipping, spot jogging	Neck rotation, shoulder rotation, waist rotation, ankle rotation, wrist rotation, spot jogging, spot sprint	Neck rotation, shoulder rotation, waist rotation, ankle rotation, wrist rotation, spot skipping, spot sprint	Neck rotation, shoulder rotation, waist rotation, ankle rotation, wrist rotation, spot jogging, spot sprint	Neck rotation, shoulder rotation, waist rotation, ankle rotation, wrist rotation, spot skipping, spot sprint
Main workout (30 min)	Lateral jump, two jump forward, one jump backward squat out, hop in jump, single leg forward hop, plank jack sprint	Push-up, shoulder tape T-rotation hand walk, push-up hold	Crunches, full sit-up, leg raise, bicycle crunches, plank or V-hold	Skipping jumping jacks, high knee, butt kick, burpee	Squat lunges, squat hold, lunge hold, donkey kick, back glute bridge, side leg raise in dog style calf raise
Cool down (15 min)	Knees hug, knees hug with cross leg, forward bend, butterfly stretch, neck side stretch, shoulder flexion, forearm stretch/wrist flexion, finger stretch and clench	Neck side stretch, shoulder flexion, forearm stretch/wrist flexion, finger stretch and clench	Cobra pose, rabbit pose, cat pose, wheel pose, knees hug, knees hug with cross leg, forward bend, butterfly stretch	Knees hug, knees hug with cross leg, forward bend, butterfly stretch, neck side stretch, shoulder flexion, forearm stretch/wrist flexion, finger stretch and clench	Knees hug, knees hug with cross leg, forward bend, butterfly stretch

Differences Between MY and PE Module

While each table contains several practices that are highly comparable to yoga practices, there are substantial distinctions between MY and PE. The intervention of MY adopts a holistic approach of body, mind, and spirit, while PE primarily focuses on the physical dimension only, though the same body postures are used sometimes.

Mindfulness-based yoga, known as mindfulness in motion, is a form of mind-body exercise promoting the well-being of the body, mind, and spirit. Through a holistic approach incorporating physical postures, breathing exercises, and meditation, the awareness toward the body, mind, and present moment is raised and sustained. The non-competitive nature of MY differs from PE, relying upon comparison to others and pushing oneself beyond limits to define progress [35]. Therefore, in the MY intervention, even though the same body positions may be used, bodily awareness, breath synchronization, and mindfulness are integrated to achieve inner balance and a sense of well-being physically, mentally, and spiritually. Apart from that, postures in MY group are performed slowly and deliberately and are often held for longer periods to dive into a deeper stretch and a wider dimension of spiritual consciousness [47].

However, PE primarily focuses on physical movement and aims at performance, fitness, and skill development. The teaching technique of PE typically is conducted with dynamism and incorporates repetitive movements, drills, and activities without any mindful awareness of breath regulation or meditation. Exercises in the PE group are usually fast-paced and dynamic, with the main goal of developing physical strength, stamina, and agility through organized physical exercises, though the intensity in this study is under control for safety and research purposes [47].

Group 3: Wait-List Control Group

Participants in the wait-list control group will continue their daily routine without receiving either MY or PE training. Three measurements will be taken at T0, T1, and T2 to compare with the other two groups. After the study period, the same MY or PE protocol will be given according to the participants' interests. The participants originally assigned to the PE group will receive MY sessions, whereas those in the yoga group will switch to the PE group. Participants in the wait-list control group can choose either class based on their personal preference after the completion of the intervention.

Blinding

This study is a single-blind RCT. In this study, outcome assessors, who are responsible for outcome measurement, will be blinded to group allocation of the participants. However, it would be hard to blind the participants given the nature of the intervention. Typically, in other research, participants would be informed of their group allocation using an opaque envelope to ensure blinding. However, given that the participants in this study are visually impaired, the opacity of the envelope is irrelevant. The single-blind design may potentially expose the study to risk of bias regarding the outcome expectations of the participants, attrition rate, response rate, and willingness to accept randomization.

Though blinding the participants will prove challenging, various strategies will be adopted to minimize the risk of participation bias stemming from expectations in case participants know their group allocation, thereby exaggerating the treatment effect. The participants will receive a reminder to refrain from revealing their group assignment to the outcome assessor at any stage of the data collection process. The research hypothesis and details of the study design will not be revealed to the participants. The informed consent form will not claim any certainty about the treatment's superiority [53]. Similar anticipated effects will be declared for both MY and PE groups, such as promoting overall well-being. Participants in both groups will receive the same number of sessions of intervention. Nondisclosure of participants' group allocation will be emphasized to the study assessor at any point in time [54]. Participants in the wait-list control group will be assured to receive either MY or PE training sessions following the same module at the end of the study according to their interests. This method has been widely used and recommended by the Evidence-Based Behavioral Medicine Committee [55].

Adherence

The research assistant will explain the study procedure clearly to make the participants understand the importance of complete attendance before the sessions start. Participants will be instructed to maintain their daily routine and not to initiate any new instructor-led exercise during the whole duration of the study. Every participant will be attentively followed up. Their assessment, as well as its health implications, will be described privately at the end of the research study by health professionals. Participants will receive certificates at the end of the research according to their performance and attendance rate.

Apart from that, all the classes will take place in the blind institutions where the visually impaired students study. Instructors will update the class attendance every day. If any absenteeism, reasons will be recorded and checked by the research coordinator. The research coordinator will intermittently observe the class without prior notification to ensure the quality of the intervention and a positive experience for the participants. After the class, feedback will be taken from participants in case of any suggestions to enhance the intervention quality.

Adverse events

Mindfulness-based yoga is generally considered safe, and negative side effects are uncommon [56]. The exercise module is also adjusted to medium intensity to reduce the risk of injury to the participants with VI. The main possible risks of both MY and PE could be muscle strains or injuries. Consequently, this program places great emphasis on performing sufficient warm-up and cool-down exercises. Props like towels, chairs, and walls will be utilized to assist participants in achieving more physically demanding postures. The yoga asanas are carefully designed, customized, and reviewed by a panel of experts so that the class will be delivered in a gradual and safe manner for those with moderate-to-severe VI. Teachers of the intervention have at least 10 years of teaching experience. Participants will be instructed to inform the principal investigator immediately, if there are any unexpected symptoms. If any complications happen during the study trial, immediate first aid will be provided, and a project adverse log will be used to record. If the problem persists, consultation with the physician will be arranged, and action will be taken as per the physician's direction. If any major serious adverse event happens, the research team will report to the Ethics Committee. Though no formal stopping rules will be set in advance, if there is any unacceptable risk of serious adverse events, the investigator will consider terminating the study.

Outcomes

The primary outcome will be the level of psychological distress assessed using the Hospital Anxiety and Depression Scale (HADS). Additionally, psychological well-being will be evaluated using the Oxford Happiness Questionnaire (OHQ), while psycho-hormonal well-being will be examined through the analysis of biomarkers such as dopamine, serotonin, cortisol, endorphin, and melatonin. The secondary outcomes will encompass psychophysiological well-being assessed by heart rate variability (HRV), electrocardiograph (ECG), galvanic skin resistance (GSR), respiratory rate (RR), and electromyography (EMG). Functional balance, mobility, and fall risk will be evaluated using the Time Up and Go test (TUG). Strength and endurance will be measured using the 30-second Chair Stand Test (30 CST). Social well-being will be assessed using the Multidimensional Scale of Perceived Social Support (MSPSS), while the Spiritual Well-being Scale (HWS), health-related quality of life will be measured by the Vision Quality and Life Index (VisQoL), and sleep quality will be assessed using the Pittsburgh Sleep Quality Index (PSQI).

Primary Outcomes

Psychological well-being in terms of anxiety and depression will be measured by the Hospital Anxiety and Depression Scale; happiness will be measured by the Oxford Happiness Questionnaire; and its related hormonal changes will be measured by dopamine, serotonin, endorphin, cortisol, and melatonin.

Hospital Anxiety and Depression Scale (HADS): Psychological well-being in terms of anxiety and depression will be measured by the HADS. This scale has been suggested for the population with VI [57]. The HADS consists of two subscales: anxiety and depression. Each subscale consists of seven items, each rated on a four-point Likert scale ranging from 0 to 3. A score of eight or above on each subscale and 15 or above on the full scale represents a clinically relevant cut-off value to detect anxiety and depressive symptoms. The English version of HADS has demonstrated satisfactory validity and reliability, with a Cronbach's alpha of 0.80 for the depression subscale and 0.81 for the anxiety subscale. The correlations between the two subscales, anxiety (HADS-A) and depression (HADS-D), varied from 0.40 to 0.74 (mean of 0.56). An optimal balance between sensitivity and specificity was achieved when the score reached eight or above on both HADS-A and HADS-D [58,59]. 

Oxford Happiness Questionnaire (OHQ): The Oxford Happiness Questionnaire will measure psychological well-being in terms of happiness. This scale has been used in the population with VI compared with the normal-sighted participants of the same age [60]. The OHQ consists of 29 items, rated by a six-point scale each (range: 1-6). It has demonstrated excellent validity and reliability with a Cronbach's alpha of 0.92 [61]. A lower score indicates a lower level of happiness. 

Biomarkers: Subjective psychological questionnaires are self-evaluated reports; objective biochemical tests have been suggested as supportive evidence from previous research to elucidate the mediating effects of MY and PE on stress and mood changes in the progression of depression and anxiety in individuals with VI [39]. Biochemical tests of dopamine, serotonin, cortisol, and endorphin are used as biomarkers to correlate with mood changes. In addition, melatonin, a neurotransmitter that regulates sleep and wakes rhythm and is closely related to psychophysiological well-being, will be assessed. Eight milliliters of blood samples will be withdrawn and centrifuged. Serum and plasma will be separated and stored at a -80-degree centigrade freezer in multiple aliquots until further processing. Higher values of dopamine, endorphin, serotonin, and melatonin indicate subjective well-being [62-64], whereas lower levels of cortisol reveal a less stressful state of mood [65]. These biomarkers indicate hormonal balance or homeostasis, which in turn affect mental and physical health [66].

Secondary Outcomes

Since psychological well-being is closely related to physiological, physical, social, and spiritual well-being, as well as health-related quality of life and sleep quality. Hence, these factors will be included as secondary outcomes.

Physiological well-being: Physiological measurement will adopt an instrument called physiography (RMS Polyrite-D) with four channels measuring heart rate variability (HRV), electrocardiography (ECG), galvanic skin resistance (GSR), respiratory rate (RR), and electromyography (EMG). Researchers will use medically graded disposable electrodes positioned on both arms and right leg to record ECG and HRV. Respiratory data will be acquired using a respiratory belt with a sensor attached. Two sensors placed on the index and middle fingers will acquire the data for GSR. The recording of EMG will be conducted with two electrodes placed on both cheeks. These variables are closely associated with autonomic tone. A higher HRV indicates less stress and a relaxed mood, while a lower value depicts stressful conditions due to a higher sympathetic tone [67]. A higher value of RR reveals stressful states such as anger and anxiety, while a lower rate of respiration indicates relaxation and well-being [68,69]. As a person becomes more stressed, GSR increases [70]. Higher EMG suggests a higher stress level [71]. Previous research has demonstrated that children with VI had significantly higher rates of breathing and heart rates than normal-sighted children of the same age [72].

Physical well-being - Time Up and Go Test (TUG Test): The Time Up and Go Test is an easily administered, cost-effective, and functionally relevant test to assess functional balance, mobility, strength, and fall risk. It was designed for older adults initially but was also used for the VI population to check their functional motor ability and balance [73]. It counts the time required for a participant to rise from a chair and sit down. Satisfactory test-retest reliability has been shown with an intra-cluster correlation coefficient (ICC) ranging from 0.8 to 0.85. Excellent concurrent validity has been demonstrated by a good correlation with the Berg Balance Scale [74].

Physical well-being - 30-second Chair Stand Test (30 CST): The 30-second Chair Stand Test (30 CST), or the 30-second Sit-to-Stand Test, is primarily designed for older adults to assess leg strength and endurance [75]. It counts the maximum number of repetitions between standing up and sitting down within 30 seconds with participants’ arms crossed in front of the chest in an armless, 17-inch-high, padded chair against a wall to maintain stability. It is a part of the Fullerton Functional Fitness Test Battery. However, it has also been used to test the population with VI for the same purpose since they tend to have less lower limb strength due to a lack of motor activity [76]. It is a simple and effective tool that provides a reliable and valid indicator of lower body strength, which is an essential factor in maintaining functional ability. The previous study has shown excellent inter-rater reliability (ICC value 0.94) and intra-rater reliability (ICC value 0.91) and has demonstrated good construct validity in assessing physical performance [77]. 

Social well-being - Multidimensional Scale of Perceived Social Support (MSPSS): The MSPSS is a self-explanatory 12-item inventory. It has good internal reliability, and test-retest reliability as well as moderate construct validity. The reliability of the whole scale was 0.88, indicating good internal consistency for the scale. The subscales (family, friends, significant other) representing the different sources of social support are strong in factorial validity. High levels of perceived social support are associated with low levels of depression and anxiety [78]. Individuals with VI tend to be socially isolated, resulting from limitations in understanding body language during communication [16].

Spiritual Well-being - Holistic Well-being Scale (HWS): The 30-item Holistic Well-being Scale (HWS) is a scale of spiritual well-being containing good psychometric properties and validity. It evaluates two core concepts of holistic well-being, which are: 1) the absence of affliction characterized by emotional vulnerability, bodily irritability, and spiritual disorientation; and 2) the presence of equanimity regarding non-attachment, mindful awareness, general vitality, and spiritual self-care. Holistic well-being focuses on the interconnectedness of body, mind, and spirit as well as spirituality, which is different from religiosity but fundamental values, beliefs, and meanings of life. Each item is a 0-10 scale ranging from "totally disagree" to "totally agree." Higher scores represent a deteriorated state of holistic well-being. [79,80].

Health-related Quality of Life - Vision Quality of Life Index (VisQoL): The Vision Quality of Life Index (VisQoL) is a short-form, six-item questionnaire used to quantify the impact of VI on the quality of life of visually impaired individuals [81]. This questionnaire assesses the following six domains: physical well-being, social well-being, emotional well-being, level of independence, self-actualization, planning, and organization. The reliability value of the Indian language version (Hindi & Telugu) exceeds 0.80. It satisfies the Rasch measurement model requirement with excellent psychometric properties as a simple summative instrument [82]. Higher scores indicate worsened QoL.

Sleep Quality - Pittsburgh Sleep Quality Index (PSQI): Pittsburgh Sleep Quality Index (PSQI) is a time-efficient self-reported sleep quality inventory to assess sleep quality and disturbance over the last month. The 19 items in the Likert-type response evaluate sleep latency, duration, and sleep disturbance [83]. It shows high test-retest reliability with Cronbach’s alpha of 0.87 and has demonstrated good validity with high correlations between PSQI and sleep log data. Sleep is considered disturbed if the value is above 5, with a sensitivity of 98.7 and a specificity of 84.4 [84]. Previous research has reported longer sleep latency and disturbed sleep cycles in individuals with VI [85].

Data collection

Prior to commencing any data collection, the data assessors will undergo training regarding the principle of data collection. The data collection procedure will be closely monitored to ensure data quality. In case of any confusion, the data assessors are directed to consult the principal investigator to clear the doubt. If any misinterpretation or misleading of the participants during the subjective psychological questionnaire interview, the data will not be considered.

Data collection at every time (T0, T1, and T2) will need approximately two weeks. Blood collection will be conducted first, followed by ECG recording, questionnaire interview, and physical test. Participants will not be occupied for more than 30 minutes daily for data collection so that their daily study routine will not be interrupted. Laboratory technicians will collect blood in the early morning from 6-9 am with fasting blood. Food and beverage will be provided once the blood collection completes for everyone as breakfast. Each day blood collection will cover around 15-20 participants so that participants do not have to wait too long with an empty stomach. The procedure will be done at the participant’s convenience to complete the follow-up. Data collection for the subjective questionnaire will be in the form of a one-to-one oral assessment, i.e., one participant and one data collector, to protect the participant's privacy.

Data collectors will confirm the time availability of the participants before the visit. The research team will get permission from the authorities before the visit for each data collection. Participants who discontinued the study intervention will be excluded. The principal researcher will keep a record of the reasons for dropping out. Once data is collected, the study investigator will enter the data with the participants’ names coded. The raw data will be stored in a locker, and only the principal investigator can access it. The research assistant will double-check the data entry in case of any mistakes.

Attrition and missing data 

Given the attrition and missing data in the experiment, the research team will account for a 20% attrition rate and closely monitor the progress of the trial with the aim in mind. To minimize attrition and missing data, it will be emphasized to both research team members and participants that full and complete participation in the intervention is crucial, regardless of the treatment assignment. Follow-ups will be scheduled at participants' convenience, with the assistance of two NGOs, and participants will receive WhatsApp reminders one day before each event.

To handle missing data, we will implement diligent monitoring and provide thorough explanations of study procedures to ensure a positive research experience. Highlighting the altruistic reasons for participation, we will elucidate the significance of maintaining involvement for all the participants. At the end of each follow-up, assessors will explain performance assessments and relevant health concerns to participants [86]. To achieve it, the research team will keenly supervise the whole procedure. The research team will contact participants through the authorities of the two NGOs for follow-up arrangements and conduct follow-up data collection at the participants' convenience.

Additionally, we will identify potential confounding factors and implement controls to mitigate their impact on the study results. This may include adjusting for baseline characteristics, using stratified randomization, and employing statistical techniques such as multivariable regression analysis to control for these factors in the data analysis.

Ethical consideration

This study trial has obtained approval from the institutional ethics committee (IEC) with the Institutional Ethics Committee Clearance Certificate Reference Number: RES/IEC-SVYASA/203/2021. It has been registered in the Clinical Trial Registration of India (CTRI) with the registration number of WHO The Clinical Trials Registry - India: CTRI/2021/08/035853.

The study procedure will be explained orally to the participants, and thumbprints will be obtained from all the eligible participants before any assessment or intervention. Apart from that, for participants aged from 15 to 18 years old, the research team will obtain oral assent with thumb print, and a consent form will be signed by their legal guardian for their willingness to allow their children to join this research program before the start of any assessment. Participation in this project will be voluntary, and participants can withdraw from the study anytime. The data will be confidential to protect the privacy of the participants. All data will be kept locked and secured, and access will be restricted to only the related research staff. Independent research assistants, without knowing group allocation, will administer the questionnaires orally to each participant and note down the answers in the hard copy. In case of doubt, the chief investigator will clarify the concerned point. Paramedical staff will withdraw blood in a standard hygienic environment. The study and ethical clearance are conducted according to the Declaration of Helsinki guidelines.

## Results

Data will be collected and extracted using the standard procedure. The appropriate statistical test will be conducted to analyze the above-mentioned variables using SPSS V. 22.0 software (IBM Inc., Armonk, New York). To find out the differences between groups, an ANOVA test will be adopted; to check within-group changes, a repeated measures ANOVA will be used. The findings will be released subsequent to the study of the data.

## Discussion

This study will enhance the current knowledge that yoga can significantly promote physical wellness for the population with VI, such as improving mobility, balance, and strength [37,38,76,87]. Previous research has shown that yoga has the ability to alleviate psychological distress for those who are experiencing continuous disequilibrium and promote their quality of life; however, it has not been extensively studied in the population with VI, according to our best knowledge [88-90]. Yoga, as a cost-effective, active self-help tool, can be used for long-term practice. This yoga program, if found effective, can be integrated into educational or rehabilitation systems to enhance the well-being of individuals with VI. This study will also focus on the implementation and reproducibility of the yoga protocol if it is widely disseminated. This study will estimate the feasibility, acceptance, and practicability of this yoga intervention as well. Apart from that, potential biases such as attrition rates, response rates, participants' acceptance of randomization, and sampling bias will be evaluated. 

This study provides an integrative approach to active self-care to empower the visually impaired population to achieve mindful awareness of their body and mind, personal transformation, and a sense of equilibrium and well-being in life. To address psychological distress among people with other disabilities and chronic illnesses, such as hearing impairment and autism, though the teaching methodology may differ, future research on yoga can adopt and utilize this MY protocol. Future studies could consider replicating the research in diverse settings or populations to further validate the generalizability of the results, though it might give different results in a different context.

Strengths

The strengths of this research are: 1) subjective psychological questionnaires correlating with objective, evidence-based assessment will strengthen the study design; 2) multiple centers with a large sample size in different geographical areas will support generating the findings of this research; and 3) a three-group study design will enhance the strength of the study. The active control group (PE group) will be compared with the experimental group (MY group) to discover the potential mechanisms of yoga, especially the effects of mindful awareness on psychological well-being. The wait-list control group will be compared with the other two to reveal the significance of either type of physical activity contributing to general well-being.

Limitations

Potential limitations and their related strategies need to be acknowledged. Subject dishonesty might happen during the interview process to protect one's privacy and social identification, as they might appear to have prosocial behaviors toward interviewers. To ameliorate the effects of bias, the interviewers will inform the participants that private information will be kept confidential, and the interview will be in the form of a one-on-one session. It is crucial to keep in mind that despite the study's design using numerous centers and a sizable sample size from various geographic regions, regional-specific factors like cultural differences, resource accessibility, and customs may still have an impact on the findings. However, in this study, stratified randomization will be adopted to minimize the potential effects of confounding factors such as location, population, educational and cultural differences where the research will take place. Since each center shares certain similarities in terms of setting, such as food habits, living standards, teaching methods, and cultural background, it is reasonable to expect that various confounding factors will have the minimum effects on the results. Additionally, this study will investigate the evaluation and implementation of the MY or PE protocol. However, social support will not be considered as one factor for randomization. Therefore, it will be possible if there will be varying levels of social support among the three groups. Dropouts and missing data may also pose a risk to internal validity. The strategy to reduce the dropout rate has been discussed previously in the section named "attrition and missing data".

To commence this research, a few challenges will be under anticipation and consideration. Implementing the interventions in various contexts may present challenges related to resource availability, training of instructors, and participant engagement. Differences in educational and rehabilitation systems could affect the ease of integration and consistency in the delivery of the interventions. Addressing these potential barriers in future research would provide more practical insights into the applicability of MY across different settings.

In addition, it is crucial to consider that the effectiveness of MY may vary based on local environmental and socio-economic conditions. Tailoring the interventions to fit specific community needs and contexts may be necessary to achieve optimal outcomes. Ensuring the long-term sustainability of the interventions after the study period ends could be challenging. Establishing strong partnerships with local institutions and securing ongoing support will be essential for the continued success of the MY program.

## Conclusions

To the best of our knowledge, this prospective study is the first attempt to trial the three-armed, multi-centered randomized controlled trial comparing the effects of mindfulness-based yoga, physical exercise, and a control group on the psychological well-being of students with early VI. The study used a strict methodology and validated outcome measures. The results are expected to not only help with creating evidence-based interventions for people with VI but also add knowledge to the body of research that shows how mindfulness-based yoga and physical exercise can improve the mental health of a wide range of people. Ultimately, the goal is to empower individuals with VI to lead fulfilling and meaningful lives by addressing their psychological needs and promoting holistic well-being. With a robust study design, this study will optimize the evidence-based research findings, enrich the knowledge of future related research, and further apply it to practice.
